# Supplementary material for: Top-down perceptual inference shaping the activity of early visual cortex
Source: Nat Commun. 2025 Nov 14;16:9998. doi: 10.1038/s41467-025-64967-x (PMC12618887; doi:10.1038/s41467-025-64967-x)
Supplement: Supplementary file 1 — Supplementary Information [file 41467_2025_64967_MOESM1_ESM.pdf]

Supplementary Information

Top-down perceptual inference shaping the activity of early visual cortex

Ferenc Csikor[1,*], Balázs Meszéna[1], Katalin Ócsai[1,2], and Gergő Orbán[1,*]

[1]Department of Computational Sciences, HUN-REN Wigner Research Centre for Physics, Budapest 1121, Hungary
[2]Department of Algebra and Geometry, Institute of Mathematics, Budapest University of Technology and Economics, Budapest 1111, Hungary
[*]Corresponding Authors. E-mail: csikor.ferenc@wigner.hun-ren.hu; orban.gergo@wigner.hun-ren.hu

Supplementary Table 1. Architecture of models with bottom-up recognition. Number of hidden units in each MLP layer computing the mean and the standard deviation of each conditional distribution in the bottom-up recognition models.

Model name	$p_\theta(\mathbf{Z}_1 \mid \mathbf{Z}_2)$	$q_\Phi(\mathbf{Z}_1 \mid \text{image})$	$q_\Phi(\mathbf{Z}_2 \mid \mathbf{Z}_1)$
shallow-VAE	N/A	(2000, 2000)	N/A
ffVAE	(2000)	(2000, 2000)	(1000, 500, 250)
shallow-VAE-50px	N/A	(3000, 3000)	N/A

Supplementary Table 2. Architecture of models with top-down recognition. Number of hidden units in each MLP layer computing the mean and the standard deviation of each conditional distribution in TDVAE models. In the non-shared model, the MLP $\mathbf{Z}_2$-ff takes its input directly from pixels, eliminating parameter sharing in the entire computation graph.

Model name	$p_\theta(\mathbf{Z}_1 \mid \mathbf{Z}_2)$	$\mathbf{Z}_1$-ff	$\mathbf{Z}_2$-ff	$\mathbf{Z}_1$-TD	$\mathbf{Z}_1$-INT
TDVAE linear integration	(1000, 2000)	(2000)	(1000, 500, 250)	(250, 500, 1000, 2000)	()
TDVAE shallow nonlinearity	(2000)	(2000)	(1000, 500, 250)	(250, 500, 1000, 2000)	(2000)
TDVAE deep nonlinearity	(1000, 2000)	(2000)	(1000, 500, 250)	(250, 500, 1000, 2000)	(2000)
TDVAE-50px shallow nonlinearity	(3000)	(3000)	(1500, 750, 500)	(500, 750, 1500, 3000)	(3000)
TDVAE-20px	(250, 500)	(500)	(250, 125, 63)	(63, 125, 250, 500)	(500)
TDVAE-20px non-shared	(250, 500)	(500)	(500, 250, 125, 63)	(63, 125, 250, 500)	(500)

Supplementary Table 3. Basic features of the trained models. Validation ELBO at the end of training and the number of active stochastic units in the trained $\mathbf{Z}_1$ and $\mathbf{Z}_2$ model layers for each presented model.

Model name	ELBO	active $\mathbf{Z}_1$ units	active $\mathbf{Z}_2$ units
shallow-VAE	-1916	1256	N/A
TDVAE linear integration	-1886	1256	27
ffVAE shallow nonlinearity	-1818	1256	25
TDVAE shallow nonlinearity	-1705	1256	199
TDVAE deep nonlinearity	-1699	1256	6
TDVAE deep nonlinearity repeated	-1699	1256	5
TDVAE deep nonlinearity untrained	$-17,319$	N/A	N/A
shallow-VAE-50px	-3110	1963	N/A
TDVAE-50px shallow nonlinearity	-2753	1963	7
TDVAE-20px	-400	314	4
TDVAE-20px non-shared	-407	314	2

Supplementary Table 4. Texture representation in the trained models. Texture family decoding accuracies from mean responses of active $\mathbf{Z}_1$ and active $\mathbf{Z}_2$ units and the number of texture-family-coding (TF-enc) and non-texture-family-coding (non-TF-enc) active $\mathbf{Z}_2$ units for all studied models. No clear distinction was found between texture-family-coding and non-texture-family-coding $\mathbf{Z}_2$ units in the ffVAE model.

Model name	$\mathbf{Z}_1$	$\mathbf{Z}_2$	TF-enc	non-TF-enc
shallow-VAE	0.107	N/A	N/A	N/A
TDVAE linear integration	0.098	0.803	4	23
ffVAE shallow nonlinearity	0.093	0.915	25	
TDVAE shallow nonlinearity	0.159	0.821	5	194
TDVAE deep nonlinearity	0.194	0.876	6	0
TDVAE deep nonlinearity untrained	0.285	0.082	N/A	N/A
shallow-VAE-50px	0.110	N/A	N/A	N/A
TDVAE-50px shallow nonlinearity	0.123	0.750	7	0
CORnet	0.130	0.977	N/A	N/A

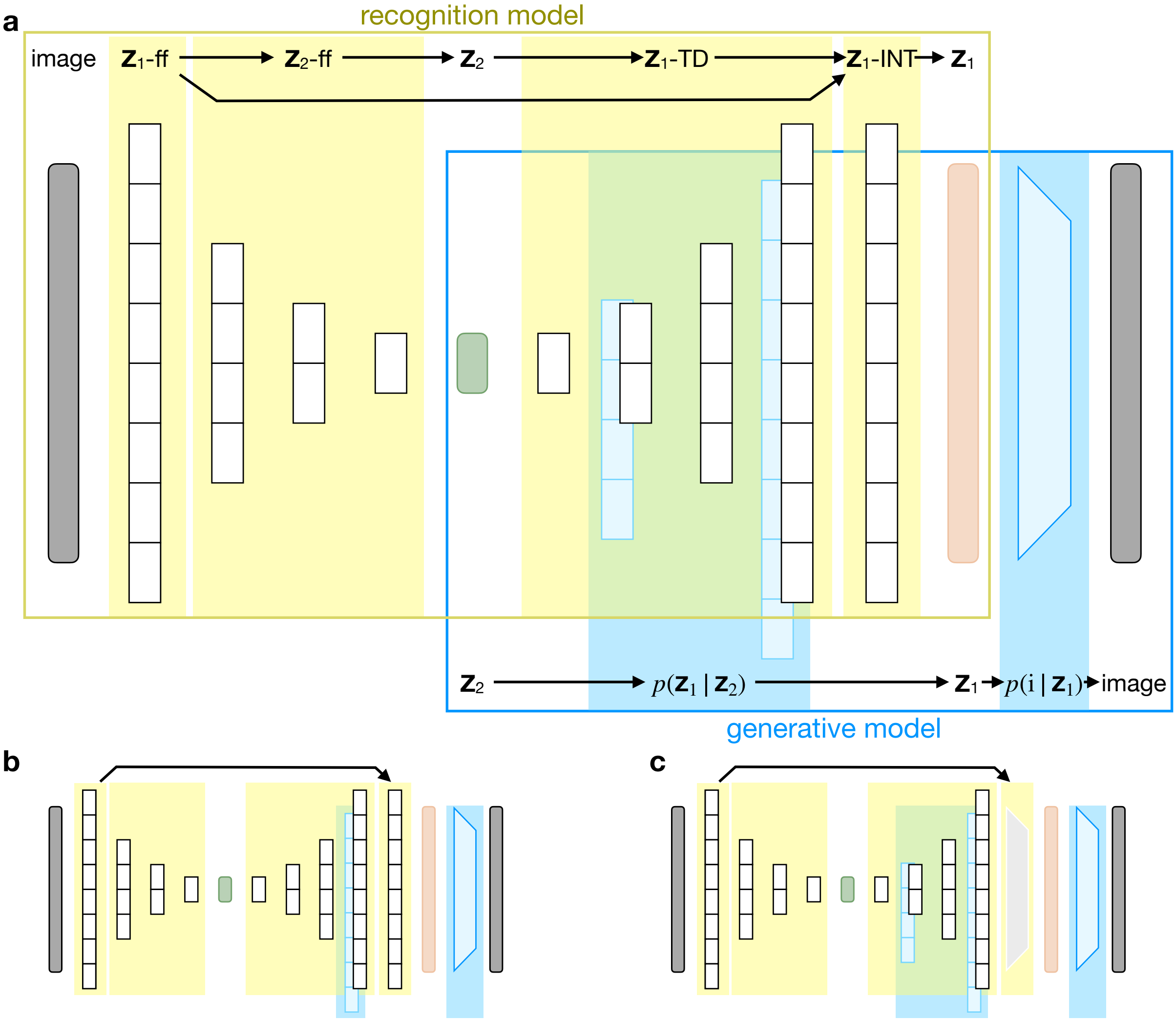

Supplementary Fig. 1. Computational graphs of TDVAE model variants. **a**, Computational architecture for our main model (TDVAE with deep nonlinearity). Each shaded object that includes multiple hidden layers represents an MLP which transforms its input in a nonlinear manner. Grey trapezoids represent linear transformations. Yellow corresponds to the recognition part of the model, blue corresponds to the generative model, MLPs of the generative model have blue shading. **b**, Computational graph of TDVAE with shallow nonlinearity. **c**, Computational architecture of TDVAE with linear integration.

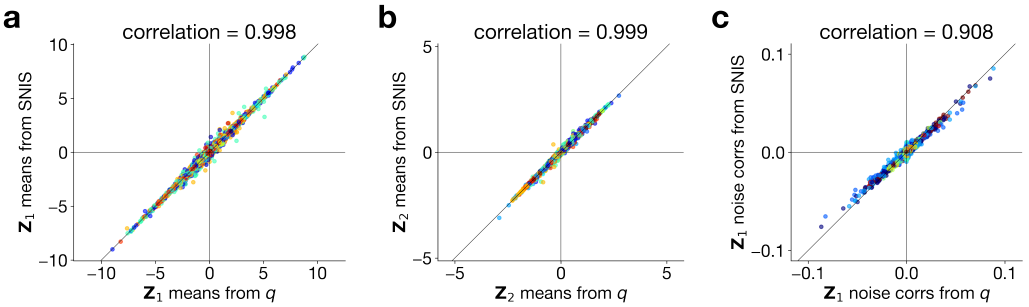

Supplementary Fig. 2. Validation of inference. Comparison of posterior means (**a**, **b**) and posterior correlations (**c**) obtained from the recognition model (denoted with q) and from the generative model (through performing self-normalizing importance sampling, *SNIS*). Moments are calculated for the 40 central, localized, medium wavelength $\mathbf{Z}_1$ units and the 6 active $\mathbf{Z}_2$ units for images sampled from the 15 texture families used throughout this paper. Individual *dots* represent means of latent posteriors (**a**, **b**) or posterior correlations of latent pairs (**c**). *Colors* represent different texture families. Moments are calculated for 80 (**a**, **b**) or three (**c**) images from every texture family. Source data are provided as a Source Data file.

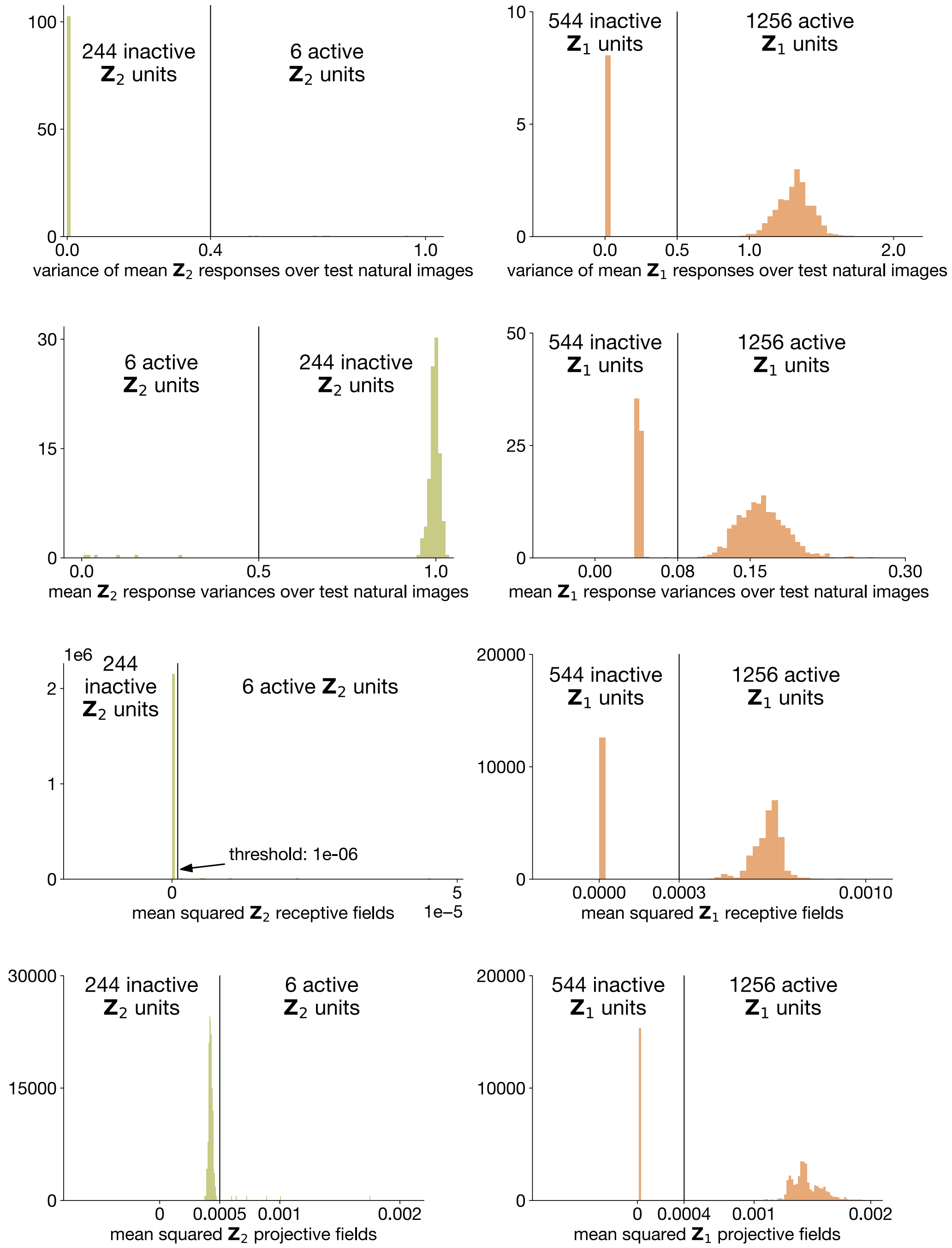

Supplementary Fig. 3. Activity of model units in the trained TDVAE model. We used four criteria for selecting active model units in the $\mathbf{Z}_1$ and $\mathbf{Z}_2$ model layers (Methods): (from top to bottom) stimulus dependent responses (variance of the mean response over the 'test' natural image dataset); response confidence (mean of the response variance over the 'test' natural image dataset); response to white noise (mean of the squared receptive field); influence on generated images (mean of the squared projective field). We found that all criteria consistently selected the same six active units in $\mathbf{Z}_2$ (left) and that, in a similar way, all criteria consistently selected the same 1256 active units in $\mathbf{Z}_1$ (right). Note that the 'training' natural image dataset has 1256 effective dimensions (Methods), meaning that the $\mathbf{Z}_1$ layer learned a complete linear basis from and over the training data. Source data are provided as a Source Data file.

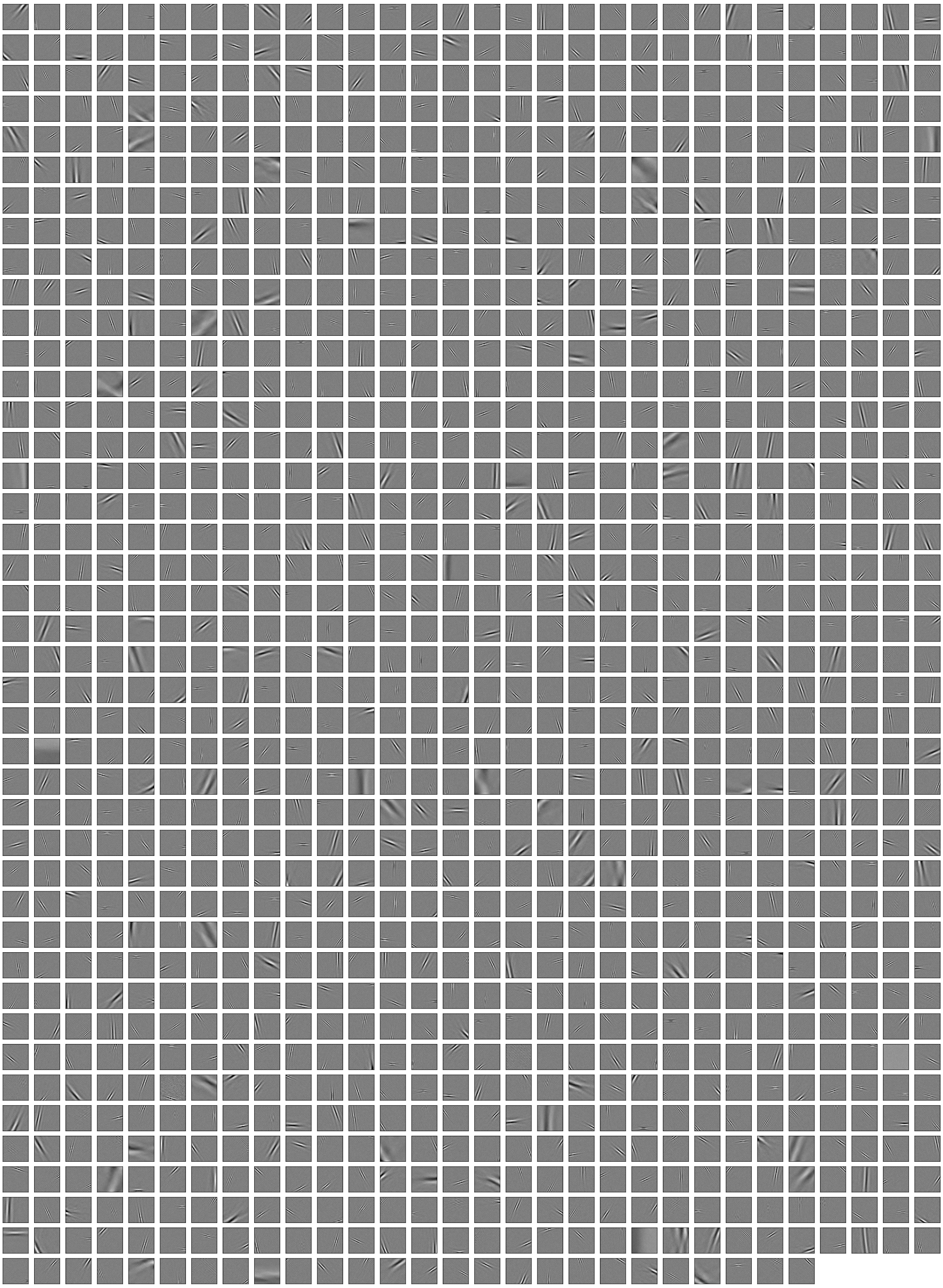

Supplementary Fig. 4. Receptive fields of all active $\mathbf{Z}_1$ units in the TDVAE model. All $\mathbf{Z}_1$ receptive fields are localized, oriented, and bandpass, reminiscent of V1 simple cells in primates.

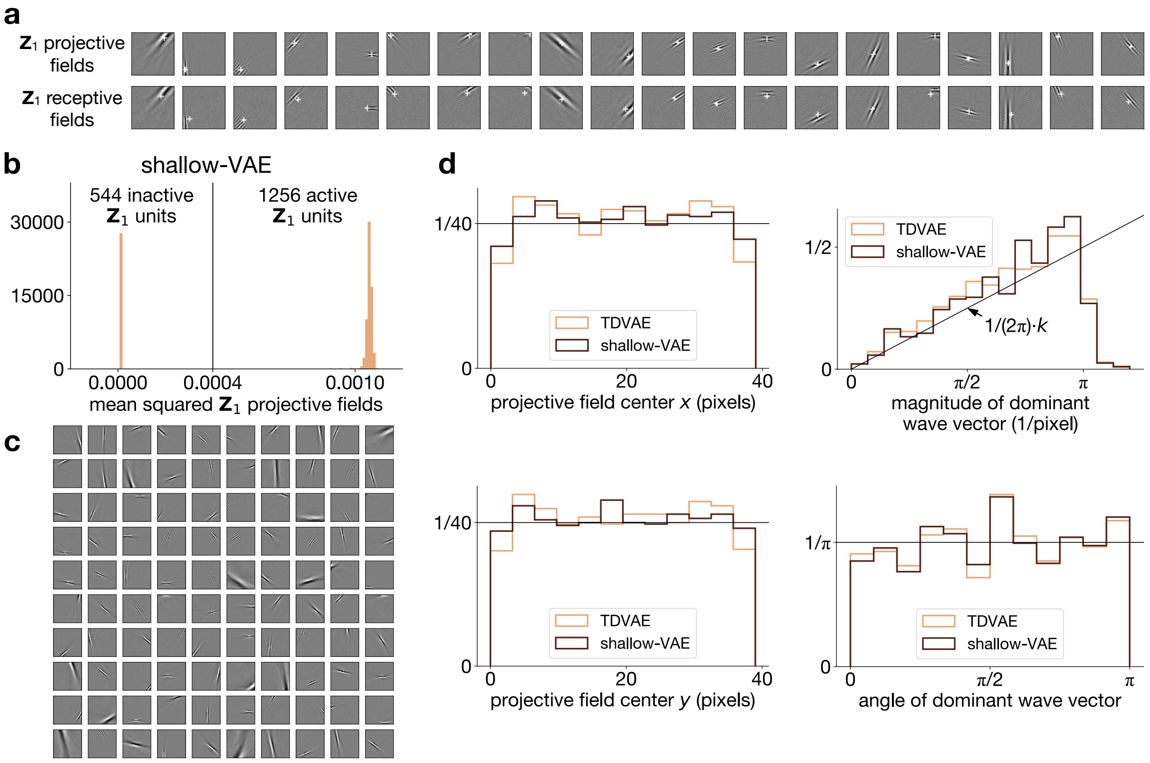

Supplementary Fig. 5. Equivalence of Z_1 receptive fields in the TDVAE and the shallow-VAE models. **a**, Example $\mathbf{Z}_1$ projective and receptive field centers calculated in the TDVAE model with the same procedure (Methods). Projective fields are very similar to receptive fields but also smoother, making them more reliable for center of mass estimation than receptive fields. In general, we use projective fields for estimating $\mathbf{Z}_1$ filter parameters throughout the paper. **b**, The shallow-VAE model has 1256 active $\mathbf{Z}_1$ units, like the TDVAE model. **c**, Example projective fields from the shallow-VAE model. These are localized, oriented, and bandpass, like those in the TDVAE model. **d**, $\mathbf{Z}_1$ projective field centers and dominant wave vectors (Methods) homogeneously fill the patch area in the TDVAE and the shallow-VAE models, making them equivalent complete bases in the space of natural training images, at least in terms of first-order probability densities. Source data are provided as a Source Data file.

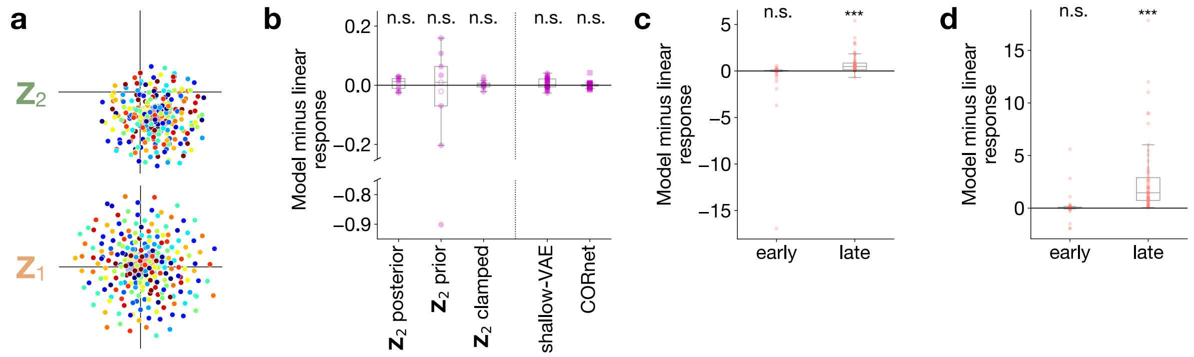

Supplementary Fig. 6. Representation and top-down effects in the TDVAE model. **a**, Two-dimensional visualization (t-SNE) of mean responses of $\mathbf{Z}_1$ and $\mathbf{Z}_2$ neurons to the texture images used in Fig. 2e in the untrained TDVAE model (*dots*). *Colors* indicate the 15 texture families as on panels Fig. 2c and 2e. *Disks*: mean across different samples from the same family. **b**, Differences between the mean and linear responses to the 'Rotated' stimulus (center line, median; box limits, upper and lower quartiles; whiskers, 1.5× interquartile range). For notation, see Fig. 4g. All t-tests are two-sided, one-sample against 0. $\mathbf{Z}_2$ *posterior*: $n = 9$ units; mean: 0.0065, $t(\text{df} = 8) = 0.92$, $p = 0.39$, 95% confidence interval $= [-0.0098, 0.23]$. $\mathbf{Z}_2$ *prior*: $n = 9$ units; mean: -0.091, $t(\text{df} = 8) = -0.85$, $p = 0.42$, 95% confidence interval $= [-0.34, 0.16]$. $\mathbf{Z}_2$ *clamped*: $n = 9$ units; mean: 0.0028, $t(\text{df} = 8) = 0.60$, $p = 0.56$, 95% confidence interval $= [-0.0080, 0.014]$. *shallow-VAE*: $n = 29$ units; mean: 0.0061, $t(\text{df} = 28) = 1.9$, $p = 0.070$, 95% confidence interval $= [-0.00053, 0.013]$. *CORnet*: $n = 40$ units; mean: 9.7×10^{-5}, $t(\text{df} = 39) = 0.070$, $p = 0.94$, 95% confidence interval $= [-0.0027, 0.0029]$. **c**, The contour completion experiment in Fig. 5d repeated with longer bars (bar lengths were 13 pixels instead of 11 pixels; center line, median; box limits, upper and lower quartiles; whiskers, 1.5× interquartile range). Only the late response shows significant boosting ($n = 57$ units in both cases; early: mean effect size: -0.40, two-sided one-sample t-test against 0: $t(\text{df} = 56) = -1.3$, $p = 0.19$, 95% confidence interval $= [-1.01, 0.21]$; late: mean effect size: 0.77, one-sided one-sample t-test against 0: $t(\text{df} = 56) = 5.5$, $p = 4.2 \times 10^{-7}$, 95% confidence interval $= [0.54, \infty]$). **d**, The contour completion experiment in Fig. 5d repeated with a larger Gaussian kernel (kernel size was 1 pixel instead of 0.9 pixels; center line, median; box limits, upper and lower quartiles; whiskers, 1.5× interquartile range). Only the late response shows significant boosting ($n = 57$ units in both cases; early: mean effect size: 0.11, two-sided one-sample t-test against 0: $t(\text{df} = 56) = 0.86$, $p = 0.40$, 95% confidence interval $= [-0.14, 0.36]$; late: mean effect size: 2.7, one-sided one-sample t-test against 0: $t(\text{df} = 56) = 6.0$, $p = 9.1 \times 10^{-8}$, 95% confidence interval $= [1.93, \infty]$). Source data are provided as a Source Data file.

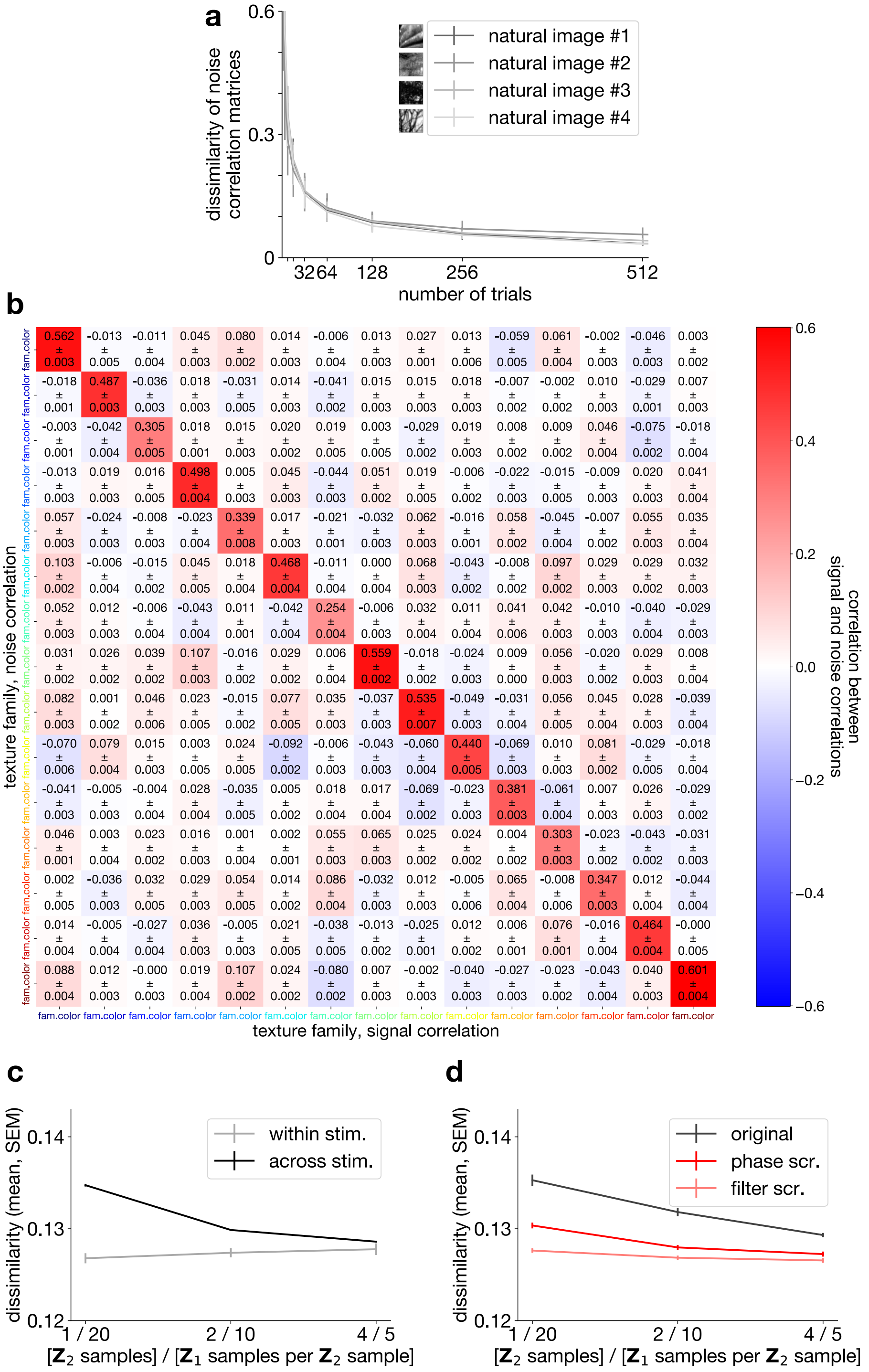

Supplementary Fig. 7. Noise and signal correlations in the TDVAE model. **a**, Estimation of noise correlations improves with the number of trials they are calculated from. Solid lines: mean within-stimulus noise correlation matrix dissimilarities for four natural image examples (*inset*). Error bars: s.d. In general, this paper presents noise correlation matrices calculated from $n = 80$ trials. **b**, Noise and signal correlations are connected through the contextual prior. Pearson correlations between noise and signal correlation coefficients over distinct pairs of $n = 40$ $\mathbf{Z}_1$ units. Correlation matrices were calculated on 15 texture families (axes), 80 samples per family, and 80 trials per sample. The whole process was repeated 5 times. Colors and numbers: means. Errors: s.e.m. **c**, Fig. 6b repeated with different model sampling settings compatible with the 400 ms recording window used in [1]. Data are presented as mean values ± s.e.m. **d**, Fig. 6f repeated with different model sampling settings compatible with the 400 ms recording window used in [1]. Data are presented as mean values ± s.e.m. Source data are provided as a Source Data file.

Supplementary References

[1] Bányai, M., Lazar, A., Klein, L., Klon-Lipok, J., Stippinger, M., Singer, W. & Orbán, G. Stimulus complexity shapes response correlations in primary visual cortex. *Proceedings of the National Academy of Sciences* **116**, 2723–2732 (2019).
