# Supplementary material for: Top-down perceptual inference shaping the activity of early visual cortex
Source: Nat Commun. 2025 Nov 14;16:9998. doi: 10.1038/s41467-025-64967-x (PMC12618887; doi:10.1038/s41467-025-64967-x)
Supplement: Supplementary file 2 — Description of Additional Supplementary Files [file 41467_2025_64967_MOESM2_ESM.pdf]

Description of Additional Supplementary Files:

Supplementary Software 1: Python source code of the TDVAE and shallow-VAE model families.
